# A possible mechanism of Cry7Ab4 protein in delaying pupation of *Plutella xylostella* larvae

**DOI:** 10.3389/fimmu.2022.849620

**Published:** 2022-09-07

**Authors:** Jing-Wen Lu, Liang Jin, Meng-Ge Li, Bryan Q. Yu, Yang-Fan Wen, Yu-Qing Gu, Yi Lin, Xiao-Qiang Yu

**Affiliations:** ^1^ Fujian Provincial Key Laboratory of Biochemical Technology, Department of Bioengineering and Biotechnology, College of Chemical Engineering, Huaqiao University, Xiamen, China; ^2^ Guangdong Provincial Key Laboratory of Insect Developmental Biology and Applied Technology, Guangzhou Key Laboratory of Insect Development Regulation and Application Research, Institute of Insect Science and Technology, School of Life Sciences, South China Normal University, Guangzhou, China; ^3^ International Department, The Affiliated High School of South China Normal University, Guangzhou, China

**Keywords:** *bacillus thuringiensis*, basic juvenile hormone-suppressible protein 1-like, Cry7Ab4, juvenile hormone, pupation delay

## Abstract

Cry toxins produced by *Bacillus thuringiensis* (Bt) are well known for their insecticidal activities against Lepidopteran, Dipteran, and Coleopteran species. In our previous work, we showed that trypsin-digested full-length Cry7Ab4 protoxin did not have insecticidal activity against *Plutella xylostella* larvae but strongly inhibited their growth. In this paper, we expressed and purified recombinant active Cry7Ab4 toxic core from *Escherichia coli* for bioassay and identified its binding proteins. Interestingly, Cry7Ab4 toxic core exhibited activity to delay the pupation of *P. xylostella* larvae. Using protein pull-down assay, several proteins, including basic juvenile hormone-suppressible protein 1-like (BJSP-1), were identified from the midgut juice of *P. xylostella* larvae as putative Cry7Ab4-binding proteins. We showed that feeding *P. xylostella* larval Cry7Ab4 toxic core upregulated the level of BJSP-1 mRNA in the hemocytes and fat body and decreased the free juvenile hormone (JH) level in larvae. BJSP-1 interacted with Cry7Ab4 and bound to free JH *in vitro*. A possible mechanism of Cry7Ab4 in delaying the pupation of *P. xylostella* larvae was proposed.

## Introduction

*Bacillus thuringiensis* (Bt) kills certain insect pests mainly due to toxic proteins generally referred to as Cry and Cyt proteins (1, 2). These Bt proteins are widely used in biopesticide formulations and transgenic crops for insect pest control (3, 4). In the alkaline environment of insect midgut, the Cry protoxin is cleaved by midgut proteases to produce an active Cry toxin (toxic core) of 60–70 kDa, which then binds to specific receptors to exert its insecticidal activity. The three-domain Cry toxin has high insecticidal toxicity against Lepidopteran, Dipteran, and Coleopteran species (5). The widely accepted mode of action of the three-domain Cry toxins is the classical pore-forming model: after activation of protoxins by midgut proteases, the active Cry toxin first interacts with potential toxin receptors, including aminopeptidase N (APN), alkaline phosphatase (ALP), and cadherin (6), and binds to midgut epithelial cells (7), then a conformational change and formation of Cry toxin oligomers result in insertion of toxins in the membrane for pore formation, which finally leads to cell lysis and death of insects (8). Another model is described as follows: after binding of the Cry toxin to receptors, intracellular signal transduction is activated to participate in the insecticidal process (9). In addition to APN, ALP, and cadherin, other Cry receptors, such as the ATP-binding cassette (ABC) transporter subfamily C2 (ABCC2) (10), α-amylase(11), and sodium solute symporter (TcSSS) (12), as well as putative Cry-binding proteins such as actin, V-ATP-synthase, flotillin, and prohibitin, have been identified or isolated from the midgut brush border membrane vesicle (BBMV) (13–15).

Our previous work has identified proteins from midgut juice that bind to Cry toxins, and these non-receptor proteins also affect insecticidal activities of Cry toxins (16–18). It has been reported that Cry toxin receptors in *P. xylostella*, including ALP and ABCC, are regulated by the MAPK signal pathway, and a high level of hormones promotes the expression of ALP and ABCC by activating the MAPK pathway, leading to Cry resistance (19). Thus, the insecticidal mechanism of Cry toxin might be more complicated than what we have already known.

Most Cry toxins at high enough concentrations can kill insects, but they may not kill insects at low concentrations. Indeed, some Cry toxins at low concentrations show inhibitory activity on insect growth. The CryIA toxin affected the growth or development of *Lymantria dispar* (20). Feeding tests with Cry1Ac and Cry1Ab showed that both toxins retarded the growth and inhibited the food intake of *Heliothis virescens* larvae (21). Bt-Cry1Ab maize and Cry1Ab13 significantly inhibited the growth and development of the survived *Spodoptera frugiperda* and *Ostrinia furnacalis* larvae, respectively (22, 23).

In our previous work, we showed that the trypsin-digested full-length Cry7Ab4 protoxin strongly inhibits the growth of *P. xylostella* larvae although it cannot kill larvae (24). Inhibition of insect growth or development by Cry toxins can contribute to controlling the population of insect pests and thus is relevant to agricultural pest control. To elucidate the mechanism of Cry toxins in inhibition of the growth or development of insect pests, in this paper, we expressed and purified recombinant active Cry7Ab4 toxic core from *Escherichia coli* for bioassay and identified its binding proteins in *P. xylostella*, as Cry7Ab4, unlike most Cry toxins, is non-lethal to *P. xylostella* larvae when added to a diet at 80 μg/g (see *Results* section). We showed that Cry7Ab4 toxic core exhibited activity to delay the pupation of *P. xylostella* larvae. Using protein pull-down assay, several proteins, including basic juvenile hormone-suppressible protein 1-like (BJSP-1), were identified from the midgut juice of *P. xylostella* larvae as putative Cry7Ab4-binding proteins. We then showed that feeding *P. xylostella* larvae with Cry7Ab4 toxic core upregulated the level of BJSP-1 mRNA in the hemocytes and fat body and decreased the level of free juvenile hormone (JH). BJSP-1 interacted with Cry7Ab4 and bound to free JH *in vitro*. A possible mechanism of Cry7Ab4 in delaying the pupation of *P. xylostella* larvae was then proposed.

## Materials and methods

### Bacterial strains and insects

*E. coli* strain DH5α (TransGen, Beijing, China) was used for gene cloning, and strain BL21 (DE3) (TransGen, Beijing, China) was used for protein expression. *P. xylostella* eggs and an artificial diet (feed formula: wheat germ, yeast, carrageenan, konjac flour, sorbic acid, vitamin C, rapeseed, rapeseed oil, sugar, 21 Gold vitamin, linoleic acid, paraben) were purchased from Henan Jiyuan Baiyun Industry Co., Ltd., China; the eggs were reared at 25 ± 2°C, 60%–70% relative humidity, and a 12 h: 12 h light cycle to second-instar larvae for bioassays.

### Preparation of recombinant Cry7Ab4 and BJSP-1

The *Cry*7 gene (GenBank accession number EU380678.1) encodes a protein (GenBank accession number ACB38747.1) named as Cry7Ab4 by *Bacillus thuringiensis* Toxin Nomenclature. To prepare Cry7Ab4 toxic core, the DNA sequence encoding Cry7Ab4 toxic core (residues 1–637) was synthesized by the DetaiBio company (Nanjing, China) and cloned into pET-32a(+) expression vector for recombinant protein expression in *E. coli* Rosetta BL21 (DE3) cells. The expression of Cry7Ab4 toxic core and Thioredoxin (Trx, control protein) was induced by addition of isopropyl-β-D-thiogalactoside (IPTG) to a final concentration of 0.5 mM, and bacterial cells were incubated at 25°C for 16 h. The supernatants of bacterial cell lysates were collected and subjected to ProteinIso Ni-NTA resins (TransGen, Beijing, China) for the purification of Cry7Ab4 toxic core and Trx according to the manufacturer’s instructions.

To prepare BJSP-1, the DNA sequence encoding the full-length basic juvenile hormone-suppressible protein 1-like (BJSP-1, GenBank accession number XM_011551310) of *P. xylostella* was synthesized by the GenScript company (Nanjing, China) and cloned into pGEX-KG expression vector for recombinant protein expression in *E. coli* Rosetta BL21 (DE3) cells. The expression of BJSP-1 and glutathione S-transferase (GST, control protein) was induced by addition of IPTG to a final concentration of 0.5 mM, and bacterial cells were incubated at 16°C for 16 h. The supernatants of bacterial cell lysates were collected and subjected to ProteinIso GST resins (TransGen, Beijing, China) for purification of BJSP-1-GST fusion protein and GST according to the manufacturer’s instructions.

### Bioassays

The activity of Cry7Ab4 toxic core was performed with second-instar day 2 P*. xylostella* larvae. Purified Cry7Ab4 toxic core in 10 mM phosphate buffered saline (PBS, pH 7.4) at increasing amounts (0, 40, and 80 μg toxin per gram diet) was added to the diet, and *P. xylostella* larvae (two larvae per Eppendorf (EP) tube, 30 larvae in each group, and three groups for each treatment) were fed a diet containing Cry7Ab4 toxic core every 24 h until pupation. The average weight of larvae and food intake were recorded and calculated every 24 h, and the difference in pupation time was recorded and analyzed.

### Protein pull-down experiments

Midgut tissue and juice from *P. xylostella* fourth-instar larvae were collected as described previously (16) and subjected to protein pull-down assays according to a described method (25). Briefly, *P. xylostella* larval midgut juice was incubated with Cry7Ab4 toxic core-coupled Sepharose™ 4B beads for 1 h at 4°C. Cry7Ab4 toxin-binding proteins were separated by SDS-PAGE and stained using FASTsilver Stain Kit (Beyotime, Jiangsu, China). This experiment was repeated at least three times. The unique protein bands were analyzed by liquid chromatography connected with tandem mass spectrometry (LC-MS/MS) at the Huada Protein Research Center (HPRC). Specific information of search engine and search parameters was the same as described previously (16).

### Homology modeling and molecular docking

Three-dimensional (3D) structures of Cry7Ab4 toxin and *P. xylostella* BJSP-1 were predicted by SWISS-MODEL (http://swissmodel.expasy.org) (26). Models were assessed using MolProbity by submitting PDB files to the MolProbity server (http://molprobity.biochem.duke.edu/) (27). Model structures of BJSP-1 and Cry7Ab4 were predicted using *Antheraea pernyi* arylphorin (PDB: 3GWJ) and *B. thuringiensis* insecticidal delta-endotoxin Cry8Ea1 (PDB: 3EB7) as templates, respectively. Docking of Cry7Ab4 toxin to *P. xylostella* BJSP-1 was carried out using ZDOCK (http://vasker.compbio.ku.edu/resources/gramm/grammx) with all default parameters (28).

### Western blot and far-Western blot analyses

For Western blot (WB) analysis, purified recombinant *P. xylostella* BJSP-1-GST and Cry7Ab4 toxic core were separated on 10% and 12% SDS-PAGE, respectively, and proteins were transferred to nitrocellulose membranes. The membrane was blocked with 5% dry skim milk in Tris-buffer saline (TBS) containing 0.05% Tween-20 (TBS-T), incubated with primary rabbit anti-GST polyclonal antibody (1:10,000) or primary mouse anti-His polyclonal antibody (1:10,000) (Proteintech Group, Chicago, USA), and then incubated with secondary alkaline phosphatase conjugated goat anti-rabbit or anti-mouse antibody (1:2,000) (Proteintech Group, Chicago, USA). Antibody binding was visualized by a color reaction catalyzed by alkaline phosphatase as described previously (29).

For far-Western blot analysis, the purified recombinant BJSP-1-GST fusion protein was separated on 10% SDS-PAGE and transferred to a nitrocellulose membrane. The membrane was washed with TBS-T, blocked with 5% dry skim milk in TBS-T at 25°C for 2 h, and then probed with purified recombinant His-tagged Cry7Ab4 toxin at 4°C overnight with gentle rocking. After washing, the membrane was then incubated with the primary mouse anti-His polyclonal antibody (1:10,000) (Proteintech, Chicago, USA) to the His-tagged probe protein, then with goat anti-mouse secondary antibody (1:1,000) (Proteintech Group, Chicago, USA), and antibody binding was detected by fluorescent signal using Azure c500 (Dublin, California, USA) (29, 30).

### Enzyme-linked immunosorbent assay

To confirm the interaction between Cry7Ab4 and BJSP-1, and BJSP-1 and free juvenile hormone (JH), a modified enzyme-linked immunosorbent assay (ELISA) microplate assay was carried out using a described method (30). Briefly, 96-well microtiter plates were coated with 8 μg/well of capture protein (Cry7Ab4 or BJSP-1-GST) at 4°C overnight. The plates were washed with PBS (pH 7.4) containing 0.05% Tween-20 (PBS-T) and then blocked with 5% dry skim milk. Protein-coated plates were incubated with BJSP-1-GST fusion protein or free JH and then washed with PBS-T. The plates were first incubated with primary rabbit anti-GST polyclonal antibody (Proteintech, Chicago, USA) and then with horseradish peroxidase (HRP)-conjugated goat anti-rabbit IgG (Proteintech, Chicago, USA) or with HRP-conjugated anti-JH antibody (Jianglaibio, Shanghai, China). Antibody binding was detected using 3,3′,5,5′-tetramethylbenzidine (TMB) substrate (Beyotime, Shanghai, China) at 450 nm on the Multiskan GO Microplate Spectrophotometer (Thermo Scientific, Massachusetts, USA). These experiments were repeated at least three times.

### Detection of free JH level in *P. xylostella* larvae

The second-instar day 2 P*. xylostella* larvae were fed diets containing increasing amounts of Cry7Ab4 toxin (from 0 to 80 μg/g) or PBS (10 mM, pH 7.4) (control); the larvae were randomly taken every 24 h (24, 48, and 72 h) into 10 mM PBS (pH 7.4) (1 mg of whole larvae in 10 μl of PBS). After homogenization, the supernatant was collected by centrifugation at 5,000 g for 5 min at 4°C. The free JH level in the supernatant was measured using the JH Assay Kit (Jianglaibio, Shanghai, China), which is based on an ELISA double-antibody sandwich method, according to the manufacturer’s instructions. This experiment was repeated at least three times.

### Quantitative real-time PCR

Total RNA was isolated from the hemocytes, fat body, and midgut of *P. xylostella* fourth-instar day 2 larvae fed a diet containing 80 μg/g of Cry7Ab4 toxic core for 24 h using RNAPure Universal RNA Plus Kit (Magen, Guangzhou, China). RNA samples were reverse transcribed to cDNA with One-Step gDNA Removal (TransGen, Shanghai, China), and quantitative real-time PCR was performed using Green qPCR SuperMix (TransGen, Shanghai, China) with FQD-48A Real-Time PCR Detection System (BIOER, Hangzhou, China). Primers used in this study are listed in **Table 1**. The conditions for qRT-PCR were as follows: 94°C for 30 s, 94°C for 5 s, 60°C for 30 s, 40 cycles. The expression of each gene was determined using the 2^−ΔΔ^CT method and normalized to elongation factor-1 alpha gene (EF-1α) (GenBank accession number: XM_011562844). All experiments were performed in triplicate, and results were plotted as the mean ± SD.

**Table 1:** qRT-PCR primers for *P. xylostella* BJSP-1 and EF1.

Primer name	Sequence (5′ to 3′)
BJSP-1 (forward)	CCGCCTCAACCACCACAACTTC
BJSP-1 (reverse)	ACCATGTCGAGCCTCTTGTCATTG
EF1 (forward)	GCCTCCCTACAGCGAATC
EF1 (reverse)	CCTTGAACCAGGGCATCT

## Results

### Feeding recombinant Cry7Ab4 toxic core delayed pupation of *P. xylostella* larvae

**Figure 1:**
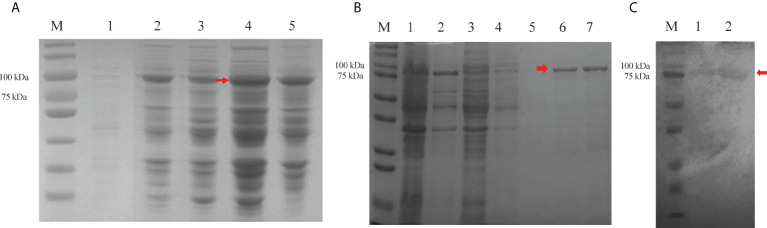
Expression and purification of recombinant Cry7Ab4 toxic core. **(A)** Expression of recombinant Cry7Ab4 toxic core in *E coli* analyzed by SDS-PAGE. M, marker; bacterial lysates without IPTG (lane 1) and with 0.5-mM IPTG induction at 25°C (lane 2) and 37°C (lane 3), respectively; supernatant (lane 4) and pellet (lane 5) from bacterial lysates with 0.5-mM IPTG induction at 25°C. **(B)** Purification of Cry7Ab4 toxic core analyzed by SDS-PAGE. M, marker; lane 1, supernatant from bacterial cell lysates containing Cry7Ab4 toxic core; lane 2, pellet from bacterial cell lysates; lane 3, flow-through from the Ni-NTA column; lanes 4 and 5, first and second imidazole (20 mM) washing fractions, respectively; lanes 6 and 7, first and second imidazole (200 mM) elution fractions, respectively. **(C)** Western blot analysis of purified recombinant Cry7Ab4 toxic core. Purified recombinant His-tagged Cry7Ab4 toxic core was separated by SDS-PAGE, transferred to nitrocellulose membrane, and detected by mouse anti-His polyclonal antibody. M, marker; lanes 1 and 2, purified recombinant Cry7Ab4 toxic core. The red arrows indicate Cry7Ab4 toxin.

**Figure 2:**
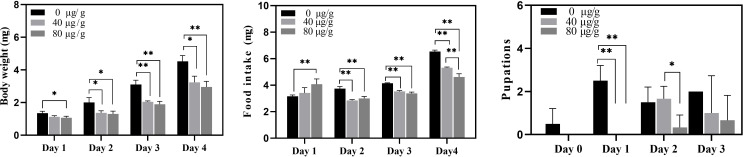
Bioassays of purified recombinant Cry7Ab4 toxic core. *P. xylostella* larvae (fourth-instar day 2) were fed diets containing 0, 40, and 80 µg/g of purified recombinant Cry7Ab4 toxic core; the average weight of larvae **(A)**, amount of diet intake **(B)**, and pupation time **(C)** were recorded after feeding Cry7Ab4 toxin. Significant difference was determined by Student’s t-test between two groups, indicated by *(*p* < 0.05), **(*p* < 0.01).

Recombinant active Cry7Ab4 toxic core (residues 1–637) was expressed in *E. coli* and purified by affinity chromatography. SDS-PAGE analysis showed that Cry7Ab4 toxic core was expressed as both soluble and insoluble products after induction with IPTG (0.5 mM) at 25°C (**Figure 1A**) and purified to homogeneity by Ni-NTA resins from the supernatant of bacterial cell lysates (**Figure 1B**). Western blot analysis showed that purified recombinant His-tagged Cry7Ab4 toxic core was recognized by anti-His antibody (**Figure 1C**). Feeding *P. xylostella* larvae a diet containing purified Cry7Ab4 toxic core inhibited larval growth (**Figure 2A**), a result in agreement with feeding larvae cabbage leaves immersed in trypsin-digested full-length Cry7Ab4 protoxin (100 µg/ml) (24), and impacted the intake of diet containing Cry7Ab4 toxin (**Figure 2B**). Moreover, feeding larvae a diet containing 40 and 80 µg/g of Cry7Ab4 toxic core delayed larval pupation by 24 h, compared to the control group (**Figure 2C**).

### Identification of Cry7Ab4-binding proteins in the midgut juice of *P. xylostella* larvae

**Figure 3:**
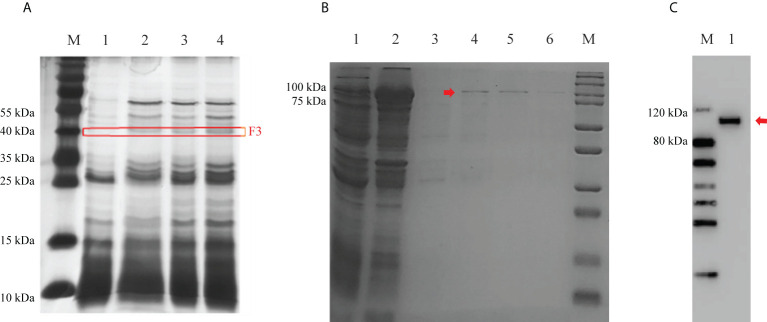
Identification of Cry7Ab4 toxin-binding proteins from the midgut juice of *P. xylostella* larvae and expression of recombinant BJSP-1-GST. **(A)** Proteins in the midgut juice of *P. xylostella* larvae that bound to Cry7Ab4 were identified by protein pull-down assay and analyzed by SDS-PAGE. M, marker; lane 1, CNBr-activated Sepharose 4B + *P. xylostella* midgut juice (control); lanes 2, 3, and 4, CNBr-activated Sepharose 4B + Cry7Ab4 (25 μl) + *P. xylostella* midgut juice (50 μl). The red box (F3) indicates protein bands of Cry7Ab4 toxin-binding proteins. **(B)** Expression and purification of recombinant *P. xylostella* BJSP-1-GST analyzed by SDS-PAGE. Lane 1, supernatant containing BJSP-1-GST from bacterial cell lysates; lane 2, pellet from bacterial cell lysates; lane 3, washing fraction (50 mM Tris–HCl, pH 8.0); lanes 4–6, first, second, and third elution fractions (50 mM Tris–HCl, pH 8.0, containing 10 mM reduced glutathione), respectively; M, marker. **(C)** Western blot analysis of purified recombinant BJSP-1-GST fusion protein. Purified recombinant BJSP-1-GST fusion protein was separated by SDS-PAGE, transferred to nitrocellulose membrane, and detected by rabbit anti-GST polyclonal antibody. M, marker; lane 1, recombinant BJSP-1-GST. The red arrows indicate BJSP-1-GST fusion protein.

To investigate the mechanism of Cry7Ab4 in delaying larval pupation, proteins in the midgut juice of *P. xylostella* larvae that can bind to Cry7Ab4 toxic core were identified by protein pull-down assay. The results showed that unique protein bands at ~40 kDa bound to Cry7Ab4 toxic core (**Figure 3A**), and the protein bands were cut out for LC-MS/MS analysis. MASCOT search results with scores higher than 100 were further analyzed by BLAST, and basic juvenile hormone-suppressible protein 1-like (BJSP-1), methionine-rich storage protein 2, apolipophorin-like, and lipase 1-like protein were identified as putative Cry7Ab4-binding proteins (**Table 2**). Among these proteins, BJSP-1 was chosen for further study.

**Table 2:** Cry7Ab4-binding proteins in the midgut juice of *P. xylostella* larvae.

MASCOT score	BLAST score		Query cover (%)		Identity (%)	Accession number	Description
517	1382		97		99.05	XP_011549612	Basic juvenile hormone-suppressible protein 1-like OS = *P. xylostella*
242	1490		97		100	BAF45386	
Methionine-rich storage protein 2					
OS = *P. xylostella*					
149	5844		99		98.59	XP_011548395	apolipophorins isoform X2 OS = *P. xylostella*
112	853		100		99.30	XP_011557736	lipase 1 OS = *P. xylostella*

### Cry7Ab4 interacted with BJSP-1

To confirm the interaction between Cry7Ab4 and BJSP-1, recombinant *P. xylostella* BJSP-1-GST fusion protein was expressed in *E. coli* and purified. SDS-PAGE analysis showed that recombinant BJSP-1-GST fusion protein was expressed after induction with 0.5 mM IPTG at 16°C for 16 h, with most BJSP-1-GST fusion protein in the insoluble fraction and some in the soluble fraction, and recombinant BJSP-1-GST was purified from the soluble fraction by affinity chromatography (**Figure 3B**). Western blot analysis showed that purified recombinant BJSP-1-GST fusion protein at ~114 kDa was recognized by anti-GST antibody (**Figure 3C**).

**Figure 4:**
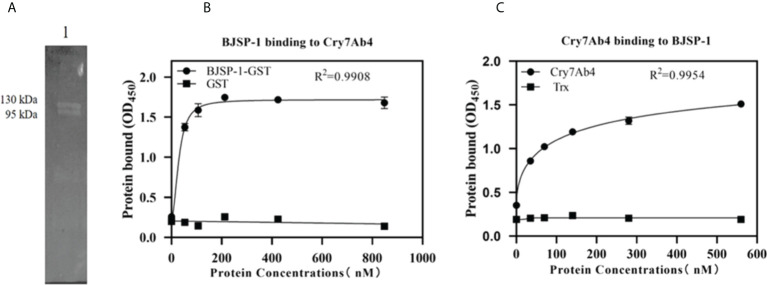
*In vitro* interaction between recombinant BJSP-1-GST and Cry7Ab4 toxic core. **(A)** Far-Western blot analysis of Cry7Ab4 binding to BJSP-1. Recombinant BJSP-1-GST was separated on SDS-PAGE and transferred to nitrocellulose membrane; the membrane was probed with His-tagged Cry7Ab4 toxic core, and binding of Cry7Ab4 to BJSP-1-GST was detected with mouse anti-His polyclonal antibody. **(B, C)** Binding of BJSP-1 to Cry7Ab4 **(B)** and Cry7Ab4 to BJSP-1 **(C)** by enzyme-linked immunosorbent assay (ELISA). Microtiter plates were coated with Cry7Ab4 toxic core **(B)** or BJSP-1-GST **(C)**; increasing concentrations of BJSP-1-GST or GST (control) **(B)** and Cry7Ab4 or Trx (control) **(C)** were added to protein-coated plates, and binding of the two proteins was detected by rabbit anti-GST polyclonal antibody to BJSP-1-GST **(B)** or mouse anti-His polyclonal antibody to Cry7Ab4 **(C)**.

The interaction between Cry7Ab4 and BJSP-1-GST was confirmed by far-Western blot analysis (**Figure 4A**) and ELISA assays (**Figures 4B, C**). Far-Western blot result showed that BJSP-1-GST fusion protein on the membrane was recognized by anti-His antibody when the membrane was probed with His-tagged Cry7Ab4 toxic core (**Figure 4A**). ELISA assays showed that when increasing concentrations of Cry7Ab4 toxic core were added to BJSP-1-coated plates, more Cry7Ab4 bound to coated BJSP-1 and the binding was saturated at 16 μg/ml of Cry7Ab4 (**Figure 4B**). Similarly, when increasing concentrations of BJSP-1-GST were added to Cry7Ab4-coated plates, more BJSP-1-GST bound to coated Cry7Ab4 and the binding was saturated at 64 μg/ml of BJSP-1-GST (**Figure 4C**). These results indicated that BJSP-1 can interact with Cry7Ab4 toxic core.

### Feeding Cry7Ab4 upregulated the expression of BJSP-1 mRNA in the hemocytes and fat body and decreased free JH level in *P. xylostella* larvae

**Figure 5:**
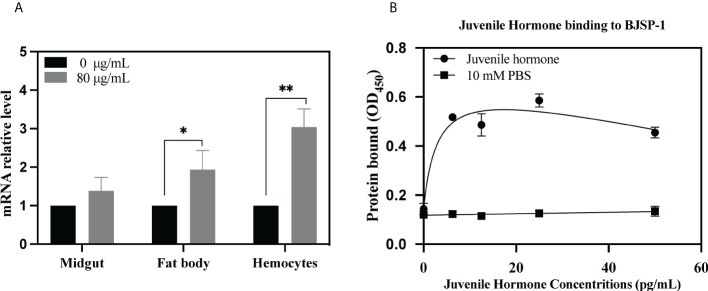
Feeding Cry7Ab4 toxin upregulated the BJSP-1 mRNA level in the hemocytes and fat body of *P. xylostella* larvae, and BJSP-1 bound to free JH. **(A)** *P. xylostella* larvae (fourth-instar day 2) were fed a diet containing Cry7Ab4 toxic core for 24 h, and the expression of BJSP-1 mRNA in the hemocytes, fat body, and midgut of larvae was determined by real-time PCR. Significant difference was determined by Student’s t-test between the control (0 μg/g of Cry7Ab4) and Cry toxin feeding groups (80 μg/g of Cry7Ab4), indicated by **(*p* < 0.01). **(B)** Microtiter plates were coated with recombinant BJSP-1-GST, increasing concentrations of free JH were added to BJSP-1-coated plates, and binding of JH to BJSP-1-GST was determined by ELISA assay and detected by horseradish peroxidase (HRP)-conjugated anti-JH antibody. *(*p* < 0.05).

As shown in **Figure 5A**, when feeding *P. xylostella* larvae (fourth-instar day 2) a diet containing 80 μg/g of Cry7Ab4 toxic core for 24 h, the level of BJSP-1 mRNA in the midgut did not change significantly; however, the BJSP-1 mRNA level was upregulated significantly in both hemocytes (3.04 vs. 1) and fat body (1.94 vs. 1).

**Figure 6:**
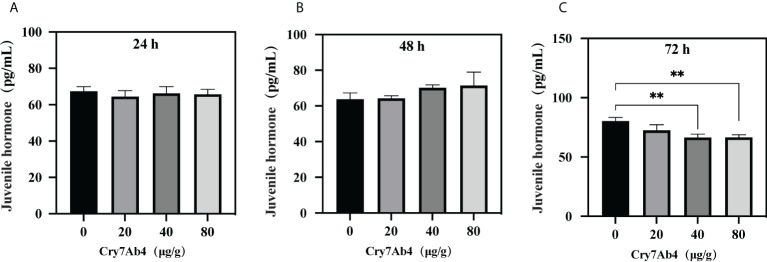
Feeding Cry7Ab4 toxin decreased the free JH level in *P. xylostella* larvae. *P. xylostella* larvae (second instar, day 2) were fed a diet containing Cry7Ab4 toxic core, and free JH level in the larvae was determined at 24 **(A)**, 48 **(B)**, and 72 h **(C)** after feeding toxin. A significant difference was determined by Student’s t-test between two groups, indicated by **(*p* < 0.01).

To determine binding of free JH to BJSP-1, ELISA assay was performed. As shown in **Figure 5B**, when increasing concentrations of free JH (from 0 to 100 pg/ml) were added to BJSP-1-coated plates, more JH bound to BJSP-1-GST and the binding was saturated at 12 pg/ml of JH. Then the free JH level in *P. xylostella* larvae after feeding Cry7Ab4 toxin core was determined. The free JH level did not change significantly after larvae were fed Cry7Ab4 for 24 and 48 h (**Figures 6A, B**); however, the free JH level decreased significantly (~16%) after larvae were fed Cry7Ab4 for 72 h (**Figure 6C**). When JH is synthesized, it immediately binds to JH-binding proteins (JHBPs) or apolipoproteins, which help deliver JH to target sites (31). Our combined results suggest that BJSP-1 may play a role in development of *P. xylostella* by binding to JH to facilitate transportation of JH.

### Molecular docking of Cry7Ab4 with BJSP-1

**Figure 7:**
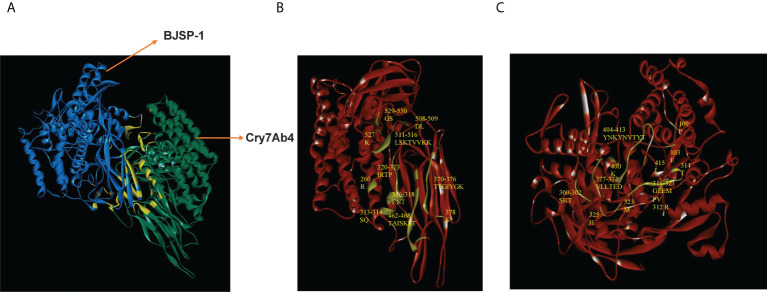
Molecular docking between Cry7Ab4 toxic core and BJSP-1. **(A)** Molecular docking of Cry7Ab4 toxic core (green) with BJSP-1 (mazarine); the binding interface is indicated in yellow. **(B)** Binding interface (prasinous) in Cry7Ab4 toxic core. **(C)** Binding interface (prasinous) in BJSP-1.

The 3D structures of BJSP-1 and Cry7Ab4 were constructed by homology modeling and accessed using the RAMPAGE server, and molecular docking between Cry7Ab4 and BJSP-1 was then performed (**Figure 7**). The result showed that the contact surface area between the two proteins was 1489 Å^2^ with a binding free energy of -15 kcal/mol. BJSP-1 interacted with Cry7Ab4 mainly through hydrogen bonds (**Table 3**) by binding to the groove formed by the three domains of toxin, mainly domain II and domain III that participate in the interaction with toxin receptors (**Figures 7B, C**).

**Table 3:** Hydrogen bonds between BJSP-1 and Cry7Ab4 from the molecular docking model.

BJSP-1	Distance [Å]	Cry7Ab4
A: LYS 301 [NZ]	2.41	B: SER 530 [OG]
A: THR 302 [N]	3.78	B: LEU 511 [O]
A: TYR 407 [N]	2.89	B: ASN 317 [O]
A: ASN 410 [ND2]	2.69	B: SER 465 [OG]
A: ASN 410 [ND2]	3.73	B: LYS 466 [O]
A: THR 302 [OG1]	3.63	B: SER 512 [N]
A: GLU 318 [O]	3.81	B: TYR 374 [N]
A: TYR 407 [OH]	3.85	B: GLN 314 [NE2]
A: TYR 407 [O]	2.26	B: THR 318 [OG1]

## Discussion and conclusion

Most insecticidal Cry proteins kill insects at the larval stage (32). Cry1Ab, Cry1F, and Cry2Aa can kill larvae at high enough concentrations; however, these toxins at low concentrations cannot kill larvae but exhibit inhibition activity on the growth or development of insects (33–35). As a novel Cry toxin found in 2008, the toxicity of Cry7Ab4 was determined against several insect pests, and the results showed that trypsin-processed Cry7Ab4 protoxin showed insecticidal activity against *Colaphellus bowringi* larvae with LC50 of 293.79 µg/ml; however, when *P. xylostella*, *Spodoptera exigua*, and *Ostrinia furnacalis* larvae were fed cabbage leaves immersed in 100 μg/ml of trypsin-processed Cry7Ab4 protoxin, Cry7Ab4 was non-lethal to larvae but inhibited larval growth (24). In this study, we showed that when *P. xylostella* larvae were fed an artificial diet containing 40 and 80 μg/g of purified active Cry7Ab4 toxic core, Cry7Ab4 was non-lethal to larvae but inhibited larval growth and delayed pupation. These results suggest that there may be different mechanisms in the mode of action between most insecticidal Cry toxins and nonlethal Cry7Ab4.

To investigate the mechanisms of Cry7Ab4 in inhibition of *P. xylostella* larval growth, we identified Cry7Ab4-binding proteins in the midgut juice of *P. xylostella* larvae and found one candidate binding protein, basic juvenile hormone-suppressible protein 1-like (BJSP-1), which belongs to hexamerins. The most abundant proteins in insect larval hemolymph are storage proteins, which are hexamerins (assembled from six ∼80-kDa polypeptide subunits). These storage proteins are synthesized mainly in the fat body and secrete into hemolymph and can reach extremely high concentrations in the last-instar larvae (36). BJSP-1 is a member of hexamerins (37), and the expression of hexamerin is increased when foreign substances are ingested by *P. xylostella* and *Galleria mellonella* (38, 39). Acidic juvenile hormone-suppressible protein 1 (AJSP-1) and BJSP-2 are highly expressed in the fourth-instar *P. xylostella* larvae and are almost undetectable in any other instar larvae (36, 40), and juvenile hormone-suppressible protein is expressed in the last-instar *Manduca sexta* larvae (41). It has been reported that in the resistant *Helicoverpa armigera* larvae, hexamerin in the gut lumen binds to Cry1Ac to block its insecticidal activity (42). We showed that *P. xylostella* BJSP-1 bound with Cry7Ab4, further supporting that non-receptor proteins like hexamerins can bind with Cry toxins.

Coincidentally, some hexamerins have been identified as JH-binding proteins (38, 43). It has been reported that when JH is combined with JHBP or apolipoprotein (both are hexamerins), it can be delivered to a specific target and exert its activity, since free JH is usually hydrolyzed by JH hydrolase (31). Here, we showed that the level of BJSP-1 mRNA was upregulated significantly in the hemocytes and fat body, and free JH was decreased in the last-instar *P. xylostella* larvae when larvae were fed Cry7Ab4 toxin. We also showed that *P. xylostella* BJSP-1 was able to bind free JH. Thus, binding of BJSP-1 to JH in the last-instar *P. xylostella* larvae may preserve the JH level for a longer time to delay pupation.

We then propose a model for the mechanism of Cry7Ab4 toxin in delaying the pupation of *P. xylostella* larvae: Cry7Ab4 toxin in the midgut somehow upregulates the expression of BJSP-1 mRNA in the fat body, and the secreted BJSP-1 protein in the hemolymph then binds to free JH to maintain the JH level in the last-instar larvae for a longer time, resulting in a delay in the pupation of larvae.

## Data availability statement

Publicly available datasets were analyzed in this study. In our previous work, a novel cry7 gene was identified in 2008 and its entire sequence has been deposited in GenBank with the Accession Number EU380678.1. The encoding protein (ACB38747.1) of this cry7 gene was named as Cry7Ab4 by Bacillus thuringiensis Toxin Nomenclature.

## Author contributions

YL and XY participated in study conception and experimental design, performed data analysis, supervised the study, and wrote the manuscript. J-WL performed most experiments, analyzed the data, and wrote the manuscript. Y-FW and Y-QG helped perform experiments. LJ, M-GL, and BY performed data analysis. All the authors read the manuscript and approved the final manuscript.

## Funding

This work was supported by the National Natural Science Foundation of China (No. 31772227), National Key Research and Development Program of China (No. 2017YFD0201201 and No. 2019YFD1002100), Key Laboratory of Biopesticide and Chemical Biology, Ministry of Education, Fujian Agriculture and Forestry University, and Fujian Key Laboratory of Ecology-toxicological Effects & Control for Emerging Contaminants (No. PY21002).

## Conflict of interest

The authors declare that the research was conducted in the absence of any commercial or financial relationships that could be construed as a potential conflict of interest.

## Publisher’s note

All claims expressed in this article are solely those of the authors and do not necessarily represent those of their affiliated organizations, or those of the publisher, the editors and the reviewers. Any product that may be evaluated in this article, or claim that may be made by its manufacturer, is not guaranteed or endorsed by the publisher.
